# Exploring the effect of different neural strategies on the knee joint contact forces during walking in adults

**DOI:** 10.1038/s41598-026-46419-8

**Published:** 2026-04-09

**Authors:** Giorgio Davico, Enrico Toccaceli, Luciana Labanca, Maria Grazia Benedetti, Marco Viceconti

**Affiliations:** 1 Department of Industrial Engineering, Alma Mater Studiorum - University of Bologna, Bologna, Italy; 2 Physical Medicine and Rehabilitation Unit, IRCCS Istituto Ortopedico Rizzoli, Bologna, Italy; 3 Department of Biomedical and Neuromotor Sciences, Alma Mater Studiorum - University of Bologna, Bologna, Italy; 4 Medical Technology Lab, IRCCS Istituto Ortopedico Rizzoli, Bologna, Italy

**Keywords:** Musculoskeletal models, Ageing, Markov Chain Monte Carlo, Joint contact forces, EMG, Computational models, Biomedical engineering

## Abstract

Identifying the different neural strategies that a person may adopt to perform simple locomotor tasks, such as walking, may enable the definition of rehabilitation plans aimed to reduce joint loads while preserving joint kinematics. Abnormal detrimental loading conditions that would likely reduce a person’s quality of life, especially among the elderly, could thus be prevented. Leveraging on previous works, we employed musculoskeletal models and biomechanical simulations (1) to explore how healthy young and elder adults recruit their muscles to perform a walking task, and (2) to determine whether the use of electromyography data to inform the simulations would allow to reduce the solution space. For the 15 tested subjects (10 young, 5 elderly), we estimated 10k sets of muscle and knee joint contact forces combining a static optimization approach with a Markov-chain Monte Carlo algorithm. We observed that the bands of solutions were narrower among the young adults than the elderly, showing how different neural strategies that prioritize the use of different muscles while ensuring the same kinematics are more likely to result in larger changes in the joint contact forces among older adults. In addition, while the neural strategies associated to the maximal knee contact forces were similar between populations, some differences emerged when analysing the strategies to minimise the knee loads. Last, the use of electromyography data allowed for a reduction of the solution band by up to 69%.

**Supplementary Information:**

The online version contains supplementary material available at 10.1038/s41598-026-46419-8.

## Introduction

The human musculoskeletal (MSK) system is intrinsically redundant: we have more actuators (muscles) than degrees of freedom (allowed motions, e.g., knee flexion). As a result, there are virtually infinite solutions one can select to perform a given motor task. Why we choose one strategy over another remains a topic of research and debate^1,2^. In a clinical context, the possibility to identify alternative muscle recruitment strategies to perform activities of daily living (ADLs) would allow for thedefinition of tailored and potentially more effective gait retraining or rehabilitation programs^3,4^. To this end, the use of MSK models and computer simulations may be key to explore the effect of different neural strategies on the resulting contact loads experienced by the joints^5–12^. However, the identification of the best approach to predict physiologically plausible solutions is not trivial.

Typically, when simulating simple and repetitive locomotor tasks, the joint contact forces (JCF) are estimated under the assumption that the MSK system works by minimizing the overall metabolic cost^13^. Although generally valid for healthy adults, this is likely not applicable to clinical populations. To overcome this limitation, Uhlrich et al.^9^ recently introduced a modified version of the classical static optimization approach in OpenSim^14,15^ where—while trying to minimize the sum of squared activations—one can prioritize the ‘optimization’ of any specific muscle by selecting the weight associated to each of them in the cost function. Alternative approaches that leverage on experimentally collected electromyography (EMG) data may allow to generate more physiologically plausible estimates^16,17^, but require data that may not be readily available. More recently, the use of muscle synergies has been proposed as a viable alternative to generate realistic predictions^18–21^. Muscle synergies are typically extracted from EMG data and carry crucial information on how different muscles work together to generate motion. In some instances, however, looking for a single ‘optimal’ solution may not be sufficient; alternative approaches to widely explore the space of feasible solutions, such as the Markov Chain Monte Carlo (MCMC) method to sample a band of possible solutions, may be preferrable^6^. The identification of suboptimal but functionally equivalent solutions may allow to get insights on motor control variability^22^.

Aiming to assess how different neural strategies affected the predicted JCFs during walking, building on^9^, Kainz and colleagues implemented a MCMC simulation framework where the individual muscle weights in the cost function varied from simulation to simulation^23^. Over 10k combinations of weights were sampled and tested, on a population of 10 children. The study highlighted the effect of different control strategies on the resulting joint loads and showed how the results may vary significantly depending on the subject and on the assigned weights. However, it is not clear whether these conclusions would apply to an adult cohort, being the latter of healthy, elder or pathological individuals. Moreover, based on the research question or clinical need, it may be necessary to find ways to reduce the complexity of the results (i.e., to narrow the band of solutions), for example by informing the simulations with EMG data. Of note, the MCMC method has recently been used for similar applications in biomechanics, as a way to extensively sample the space of solutions^24–28^.

Therefore, we set out to study how different neural strategies predicted via MSK modelling and biomechanical simulations may affect the loads experienced by the knee joint in an adult population walking on level ground. The knee joint plays a key role in many ADLs and is spanned by many bi-articular muscles whose activation impacts on multiple joints of the lower limb^8^, making it an ideal candidate for a computational study. Even more so if one considers (1) that the ageing process is associated to changes in neuromuscular control^29–31^, and (2) that abnormal knee joint contact forces are commonly observed in clinical populations, such as patients with knee osteoarthritis^32,33^ or people with a total knee arthroplasty^34–36^, due to altered muscle activations.

Thus, the aims of this study were twofold. First, to test whether the MCMC method proposed by Kainz and colleagues highlighted any differences in the bands of JCFs predicted by the models between healthy young and elderly subjects performing a simple locomotor task. Second, to test whether the use of EMG data to inform the simulations would lead to a significant narrowing of the solutions band.

## Results

### Effect of EMG data on the predictions

**Fig. 1:**
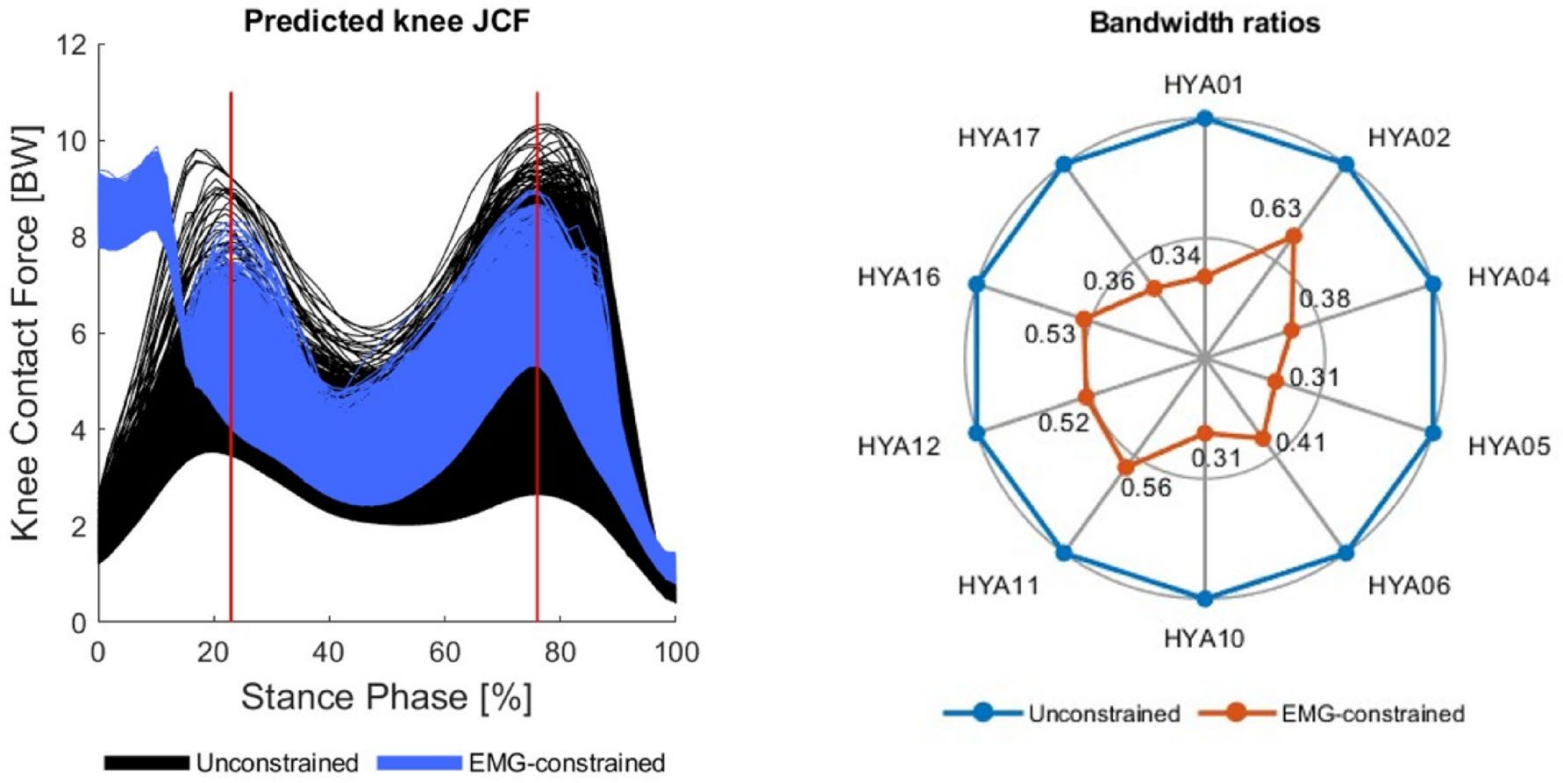
Example of knee joint contact forces predicted via unconstrained (black) and EMG-constrained (blue) simulations for one healthy young adult (HYA). In white, the reference solution resulting from setting all weights to 1 (traditional static optimization). The red lines represent the instants corresponding to the peak vertical ground reaction forces. On the right, a radar plot showing for all HYA subjects the ratio between the solution bandwidth of the unconstrained simulations (reference) and the EMG-constrained simulations.

In general, for the healthy young cohort, the use of experimental EMG data to inform and constrain the simulations was associated with a markedly reduced variability in the predicted knee JCFs (Fig. 1). The solution band was significantly narrower for the *EMG-constrained* (than for the *unconstrained*) simulations, with reductions ranging from approximately 37% (HYA02) to 69% (HYA10) (Fig. S1, Supplementary Files).

Notably, in most cases, the *EMG-constrained* simulations produced significantly larger knee JCF estimates than the corresponding *unconstrained* simulations (i.e., when the same set of weights was applied). This was not true for two subjects, HYA01 and HYA17, for whom most of the simulations were associated with a negative relative area difference (RAD), which indicate a larger area under the curve in *unconstrained* rather than *EMG-constrained* simulations (Table 1).

**Table 1:** Relative area difference (RAD) between the knee joint contact forces predicted via unconstrained and EMG-constrained simulations.

Subject ID	Relative area difference (RAD)			
	Increase		Decrease	
	Mean diff. (%)	*N*° simulations	Mean diff. (%)	*N*° simulations
HYA01	14.6 ± 8.6	4386	16.4 ± 8.1	5614
HYA02*	28.4 ± 20.6	7353	10.3 ± 7.3	2647
HYA04*	27.1 ± 16.5	5608	17.0 ± 9.7	4392
HYA05	25.4 ± 16.5	5100	23.9 ± 13.2	4900
HYA06	29.3 ± 18.5	5409	21.0 ± 11.3	4591
HYA10	26.8 ± 15.6	5178	20.7 ± 11.1	4822
HYA11*	29.3 ± 21.6	7052	11.6 ± 8.2	2948
HYA12*	33.0 ± 24.0	6674	10.7 ± 6.7	3326
HYA16*	39.9 ± 29.1	7610	9.8 ± 7.5	2390
HYA17	9.9 ± 7.6	3373	23.2 ± 12.7	6627

Differences are reported, for each participant, as percent values, where ‘Increase’ means larger area under the curve for the *EMG-constrained* simulation and ‘Decrease’ means larger area under the curve for the *unconstrained* simulation. The number of simulations (out of 10000) for which either an increased or decreased RAD value was observed is reported for completeness. Stars indicate the subjects for whom *EMG-constrained* simulations returned JCFs significantly larger than *unconstrained* simulations (Wilcoxon signed rank test, *p* = 0.05).

### Joint contact force solution bands—differences between populations

**Fig. 2:**
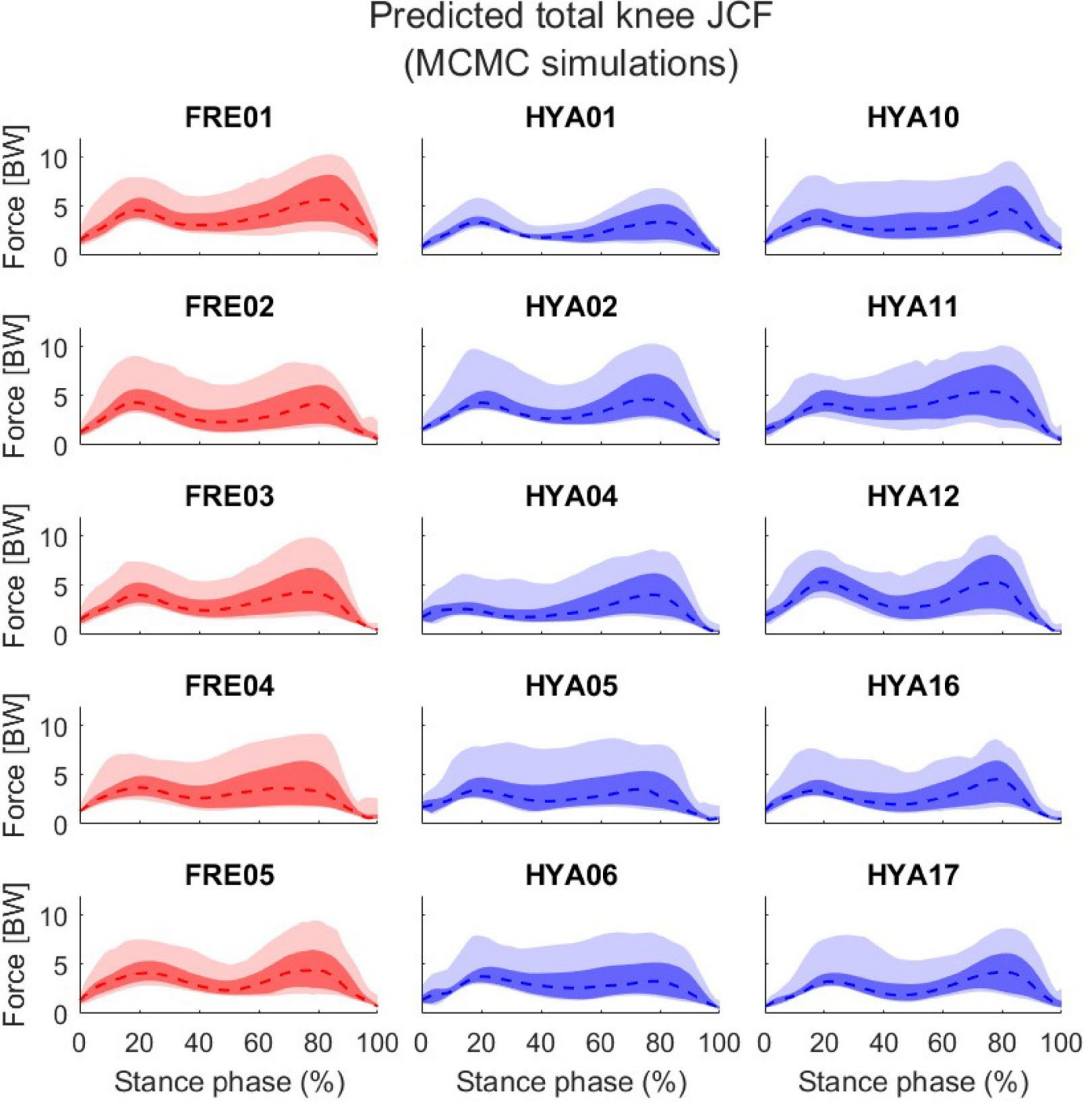
Knee joint contact forces resulting from the unconstrained simulations. Values are normalized to each subjects’ bodyweight. The entire solution bands are reported in light colours. Darker shades refer to the 10th to 90th percentiles. The dashed lines correspond to the median solutions.

**Fig. 3:**
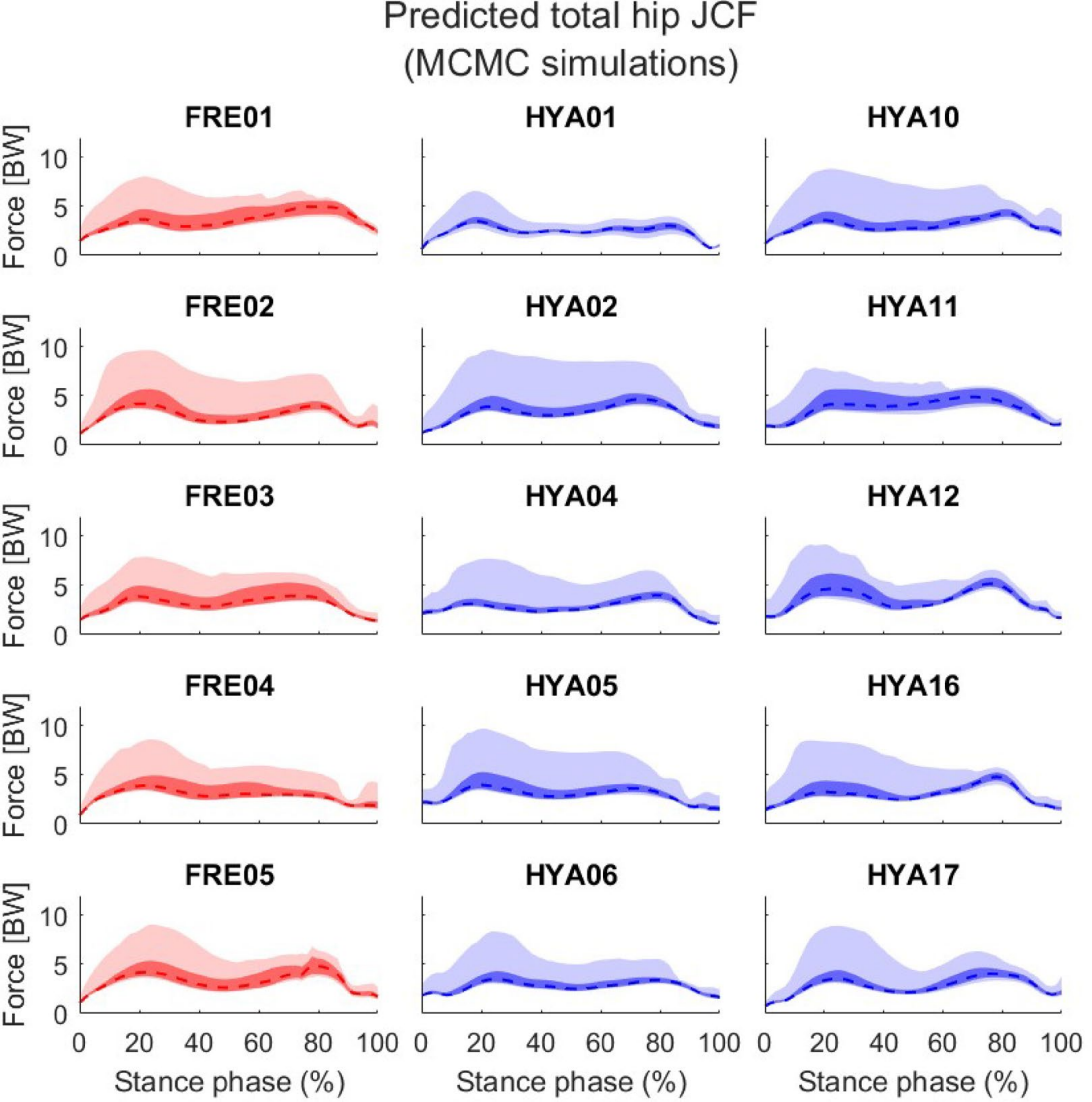
Hip joint contact forces resulting from the unconstrained simulations. Values are normalized to each subjects’ bodyweight. The entire solution bands are reported in light colours. Darker shades refer to the 10th to 90th percentiles. The dashed lines correspond to the median solutions.

**Fig. 4:**
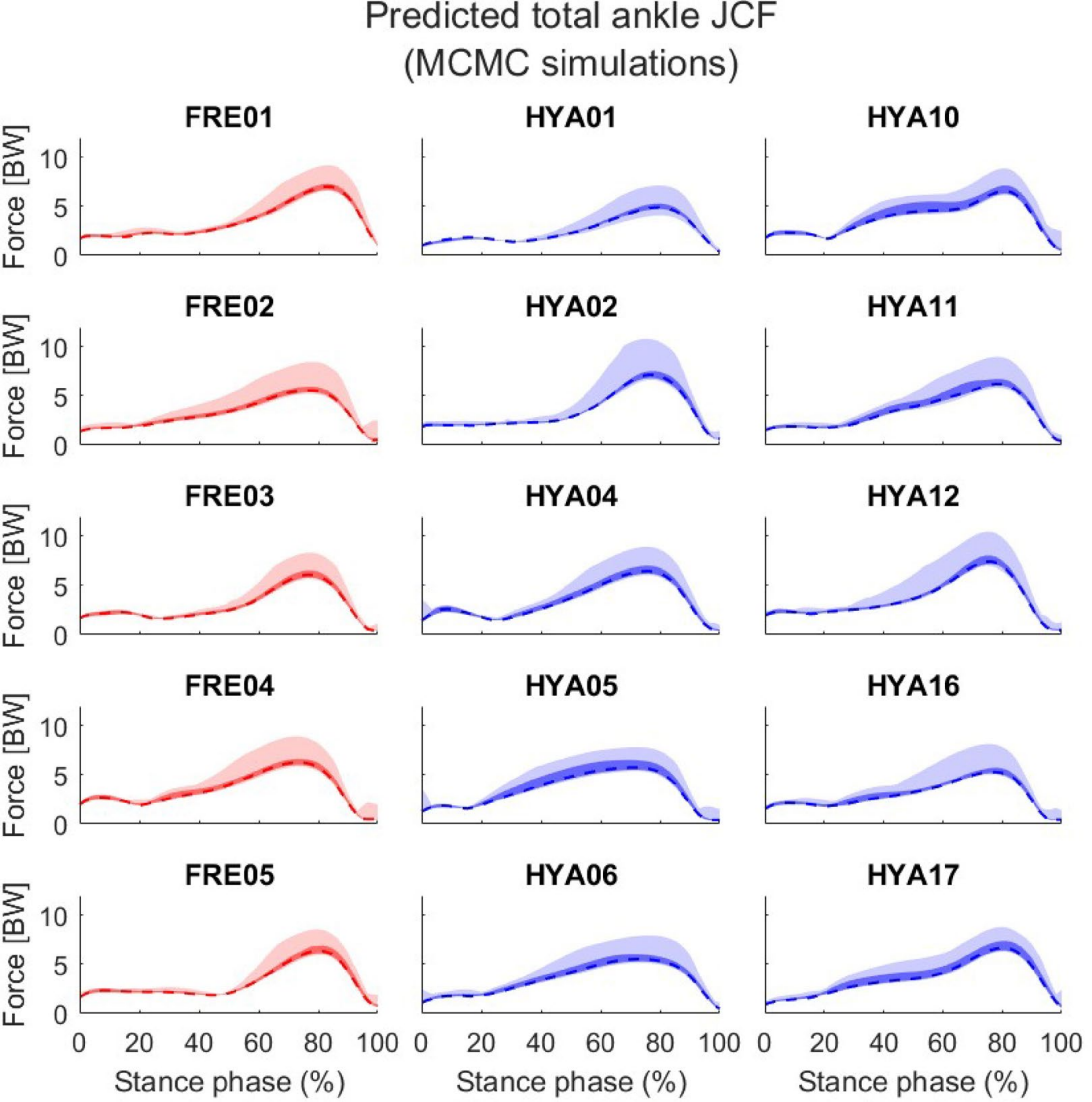
Ankle joint contact forces resulting from the unconstrained simulations. Values are normalized to each subjects’ bodyweight. The entire solution bands are reported in light colours. Darker shades refer to the 10th to 90th percentiles. The dashed lines correspond to the median solutions.

On average, the elderly participants showed a wider variability in the resultant knee JCFs compared to the healthy young cohort, as highlighted by a slightly larger solution bandwidth both in correspondence of the two characteristic peaks (initial contact and push off phases, Table 2) and across the entire stance phase (4.88 vs. 4.74 BW), when *unconstrained* simulations were performed (Table 3; Fig. 2). A similar trend was observed at the hip joint, while the ankle JCF solution bandwidth resulted to be slightly larger among the younger participants than the elderly. The above differences were more apparent when looking at the 10th -90th percentiles (Figs. 2, 3 and 4), although the solution bands were much narrower (Table 3).

**Table 2:** Knee joint contact force solution bandwidth, for the elderly (FRE) and young participants (HYA).

HYA01	1st peak	2nd peak
	3.03 [2.81,5.83]	5.55 [1.25,6.80]
HYA02	5.84 [3.49,9.33]	7.59 [2.64,10.24]
HYA04	4.24 [1.95,6.20]	6.64 [1.81,8.44]
HYA05	5.31 [2.55,7.86]	6.62 [1.61,8.23]
HYA06	4.76 [2.95,7.71]	6.41 [1.79,8.20]
HYA10	5.10 [2.83,7.92]	7.10 [2.31,9.40]
HYA11	4.26 [2.80,7.05]	8.19 [1.82,10.01]
HYA12	4.73 [3.76,8.49]	8.05 [2.05,10.10]
HYA16	4.62 [2.74,7.36]	6.90 [1.67,8.57]
HYA17	5.13 [2.75,7.88]	6.68 [1.97,8.65]
FRE01	4.44 [3.53,7.97]	7.79 [2.47,10.26]
FRE02	5.83 [3.25,9.10]	6.57 [1.75,8.31]
FRE03	4.47 [2.94,7.41]	8.00 [1.90,9.90]
FRE04	4.65 [2.48,7.13]	7.34 [1.71,9.05]
FRE05	4.60 [2.84,7.45]	7.56 [1.88,9.44]
Avg HYA	4.70 ± 0.76	6.97 ± 0.80
Avg FRE	4.80 ± 0.58	7.45 ± 0.55

All values, computed in correspondence with the two characteristic peaks of the gait cycle, are expressed in bodyweights (BW). Minimum and maximum values are reported in brackets.

**Table 3:** Hip, knee and ankle solution bandwidths.

	All solutions			90^th^ percentiles		
	Hip	Knee	Ankle	Hip*	Knee	Ankle
HYA01	1.90 ± 0.89	2.93 ± 1.37	1.30 ± 1.12	0.43 ± 0.20	1.56 ± 1.09	0.23 ± 0.16
HYA02	4.53 ± 1.67	5.05 ± 1.81	1.70 ± 1.50	0.90 ± 0.34	2.03 ± 1.21	0.32 ± 0.25
HYA04	3.15 ± 1.22	4.48 ± 1.51	1.56 ± 0.96	0.59 ± 0.16	2.01 ± 1.21	0.52 ± 0.21
HYA05	4.04 ± 1.63	5.36 ± 1.80	1.75 ± 0.82	0.91 ± 0.44	2.23 ± 1.13	0.71 ± 0.37
HYA06	2.89 ± 1.29	4.87 ± 1.49	1.69 ± 0.89	0.66 ± 0.24	2.16 ± 1.08	0.52 ± 0.21
HYA10	4.01 ± 1.42	5.35 ± 1.31	1.63 ± 0.83	0.81 ± 0.28	2.31 ± 1.00	0.70 ± 0.31
HYA11	2.81 ± 1.09	5.55 ± 1.85	1.65 ± 1.06	1.37 ± 0.56	2.85 ± 1.63	0.55 ± 0.28
HYA12	2.91 ± 1.46	4.88 ± 1.70	1.87 ± 1.19	1.07 ± 0.66	2.68 ± 1.55	0.40 ± 0.22
HYA16	3.37 ± 1.68	4.44 ± 1.41	1.63 ± 0.95	0.78 ± 0.38	2.04 ± 1.09	0.40 ± 0.16
HYA17	3.16 ± 1.62	4.46 ± 1.54	1.74 ± 0.75	0.71 ± 0.31	1.79 ± 1.00	0.69 ± 0.29
FRE01	3.04 ± 1.17	5.11 ± 1.49	1.31 ± 0.94	1.27 ± 0.44	2.50 ± 1.12	0.36 ± 0.14
FRE02	4.18 ± 1.47	5.05 ± 1.37	1.79 ± 1.02	0.97 ± 0.48	2.38 ± 0.92	0.37 ± 0.13
FRE03	3.12 ± 1.12	4.66 ± 1.89	1.18 ± 0.93	1.25 ± 0.54	2.25 ± 1.15	0.36 ± 0.20
FRE04	3.27 ± 1.16	5.05 ± 1.80	1.67 ± 1.02	1.01 ± 0.50	2.44 ± 1.27	0.46 ± 0.17
FRE05	3.27 ± 1.33	4.56 ± 1.58	1.20 ± 0.94	1.16 ± 0.46	2.06 ± 0.92	0.37 ± 0.25
Avg HYA	3.27±0.76	4.74±0.75	1.65±0.15	0.82±0.26	2.17±0.38	0.50±0.17
Avg FRE	3.38±0.46	4.88±0.26	1.43±0.28	1.13±0.14	2.32±0.18	0.38±0.04

Values are reported in units of bodyweight (BW), and represent the individual mean and standard deviation across the stance phase of the gait cycle for each elder (FRE) and young participants (HYA). *p < 0.05 (between populations).

**Fig. 5:**
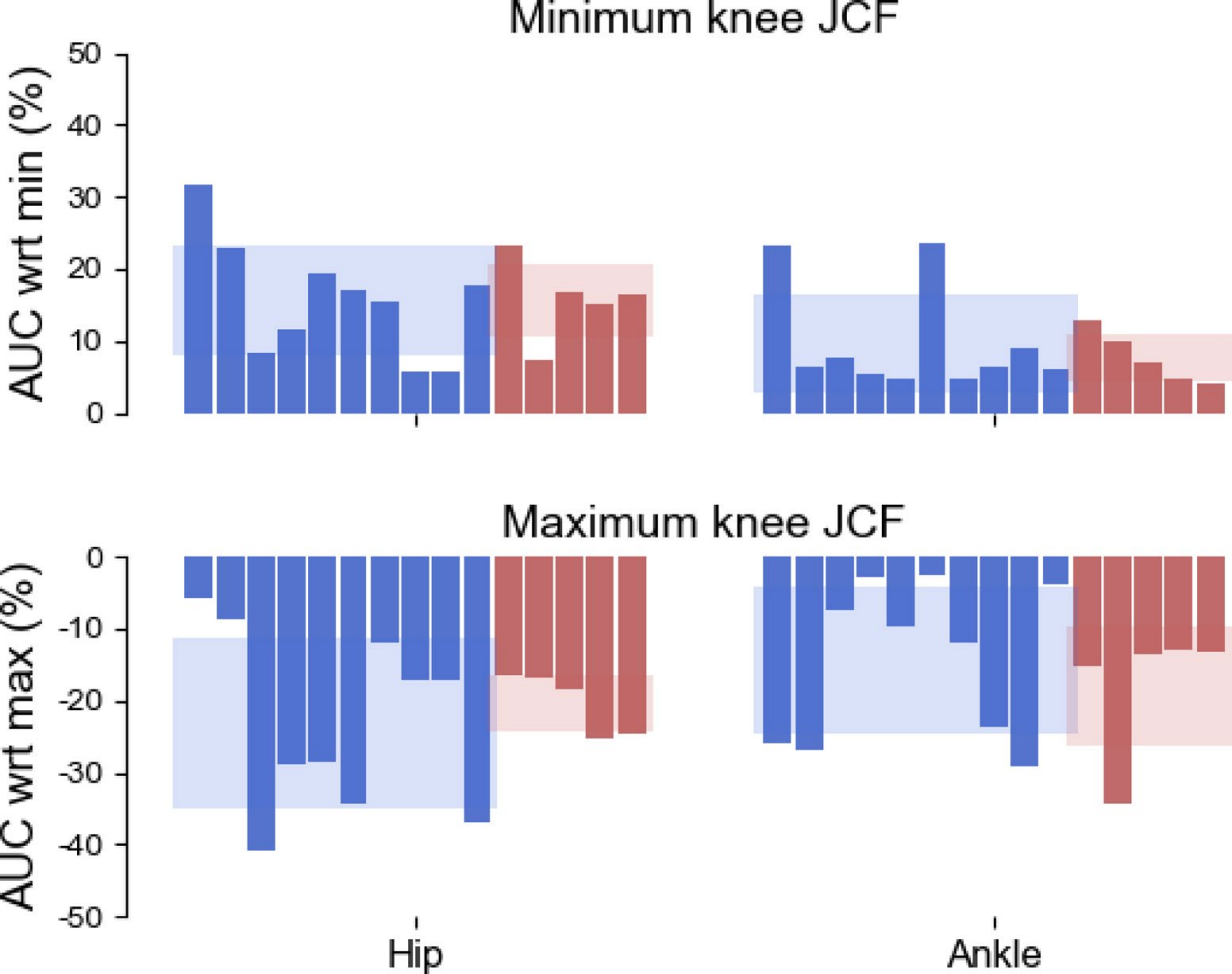
Effect of maximising or minimising the knee JCFs on the hip and ankle loads. Barplot showing the percent difference with respect to the minimal or maximal area under the hip and ankle joint contact forces, for the solutions resulting in the minimal and maximal knee JCF. The shaded areas represent the mean and standard deviation across the two populations (blue = young, red = elderly). AUC = area under the curve, JCF = joint contact force, wrt = with respect to.

In general, the solutions resulting in the minimal area under the knee JCF curve were associated to non-minimal hip and ankle JCFs, whose areas under the curve were on average 15.6 ± 6.9% and 9.0 ± 6.0% larger than their corresponding minimum among the 10k tested solutions (Fig. 5). On the other end, the solutions resulting in the maximal area under the knee JCF curve were associated to sub-maximal hip and ankle JCFs. The latter were respectively smaller than 22.1 ± 10.0% and 15.5 ± 9.8% compared to the maximal area under the solutions in the MCMC band. In both cases, the differences between populations were little.

### Comparison between the reference solution and the solutions associated to minimum and maximum knee loads

**Fig. 6:**
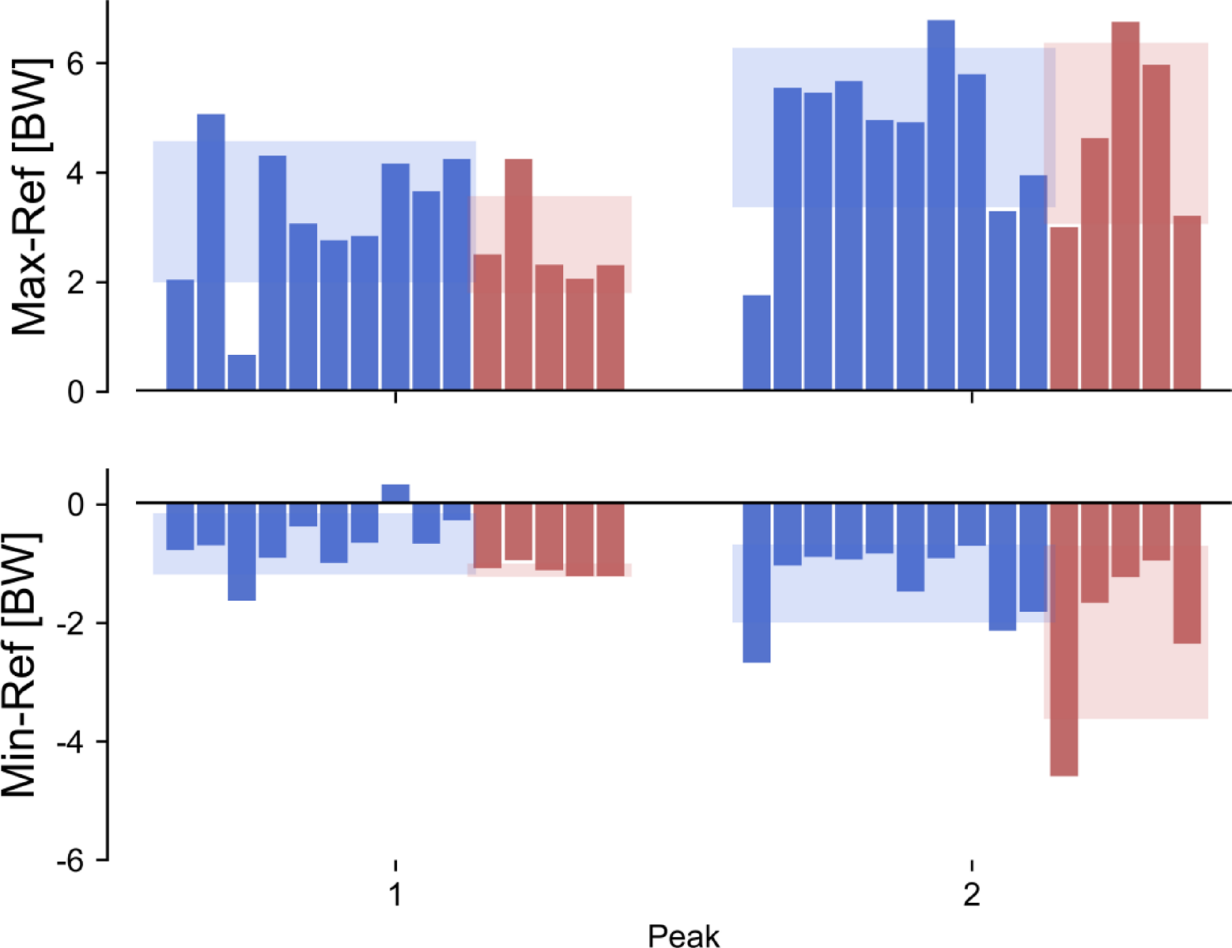
Barplot showing the difference between the solutions resulting in maximal (Max) and minimal (Min) knee joint contact force and the reference static optimization solution (Ref), among the young (HYA, blue) and elderly (FRE, red) participants. The shaded areas represent the mean and standard deviation across the two populations (blue = young, red = elderly). All values are expressed in bodyweights (BW).

In general, in correpsondence of the two characteristic peaks of the knee JCF profiles, the solutions associated to the maximal area under the curve were larger than the corresponding reference solution from static optimization (Fig. 6). This was observed across all participants, and was more apparent in correspondence to the second peak ($Diff_{{HYA}}^{{1st}}=~3.28 \pm 1.29~BW$; $Diff_{{FRE}}^{{1st}}=2.69 \pm 0.89~BW;~Diff_{{HYA}}^{{2nd}}=~4.82 \pm 1.45~BW$; $Diff_{{FRE}}^{{2nd}}=4.71 \pm 1.65~BW$). By comparison, the differences between the solutions associated to the minimal area under the curve and the corresponding reference solutions were smaller in magnitude, and larger across the elderly ($Diff_{{FRE}}^{{1st}}=~ - 1.11 \pm 0.11~BW$; $Diff_{{FRE}}^{{2nd}}= - 2.16 \pm 1.46~BW)$ than the young participants ($Diff_{{HYA}}^{{1st}}=~ - 0.66 \pm 0.51~BW$; $Diff_{{HYA}}^{{2nd}}= - 1.34 \pm 0.66~BW)$. For one participant (HYA12), the reference solution was lower than the solution corresponding to the minimal area under the curve, in correspondence of the 1st peak.

**Fig. 7:**
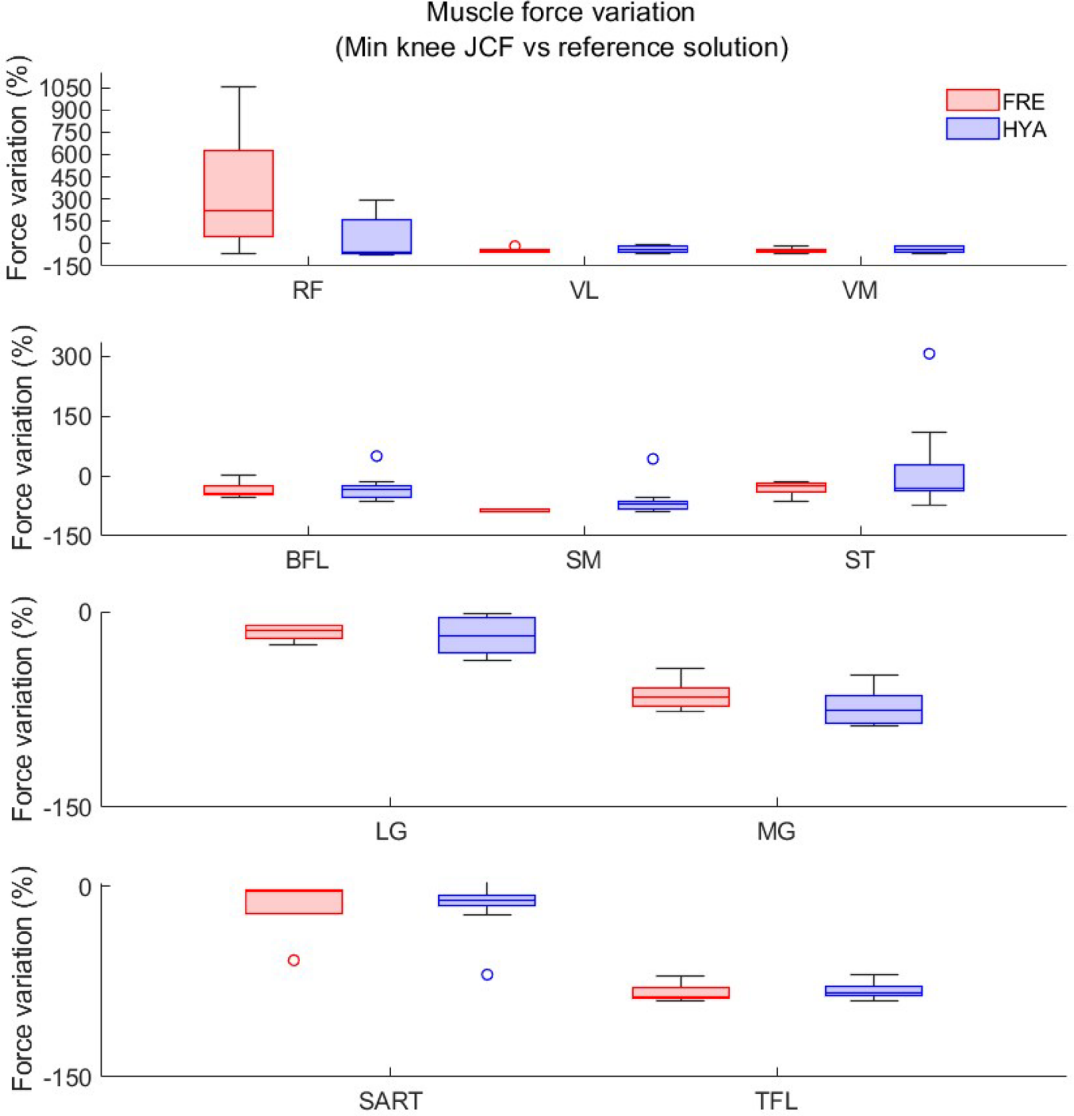
Boxplot showing the percent muscle force variation between the reference static optimization solution and the solution resulting in minimal knee joint contact forces, for the primary muscles spanning the knee joint among the young (HYA, blue) and elderly (FRE, red) participants. The reference solution corresponds to the solution obtained by setting all muscle weights to 1 (standard static optimization objective function).

In both populations (young and elderly), among the MCMC *unconstrained* solutions, the minimal area under the knee JCF curves typically resulted from a set of reduced (sometimes even zeroed, i.e. -100% variation) muscle forces compared to the reference static optimization solution (where all weights in the objective function were set to 1)—both for the knee flexor and extensor muscles. In a few cases, however, the activity of the hamstrings (among young participants) and of the RF muscle (among the elderly) increased (Fig. 7, Figure S2—Supplementary Files). Notably, the general reduction in the force generated by the muscles spanning the knee joint, which was observed in ~ 75% of the cases both among the elderly and the young adults, was compensated by an increase in the activity of the primary muscles acting on the hip (e.g., adductor muscles and gluteii. Figure S3, Supplementary Files).

**Fig. 8:**
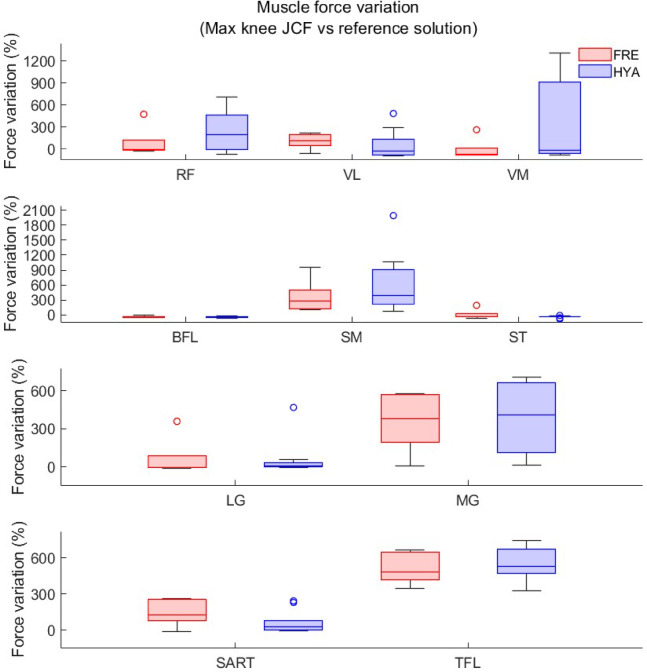
Boxplot showing the percent muscle force variation between the reference static optimization solution and the solution resulting in maximal knee joint contact forces, for the primary muscles spanning the knee joint among the young (HYA, blue) and elderly (FRE, red) participants. The reference solution corresponds to the solution obtained setting all muscle weights to 1 (standard static optimization objective function). Similarly. but opposite, the largest knee JCFs in the solution band were the result of a substantial increment, (up to 2000%) in the force developed by several muscles crossing the knee joint, (Fig. 8, Figure S2&S4—Supplementary Files), compared to the default static optimization solution. Differences between populations were little.

**Fig. 9:**
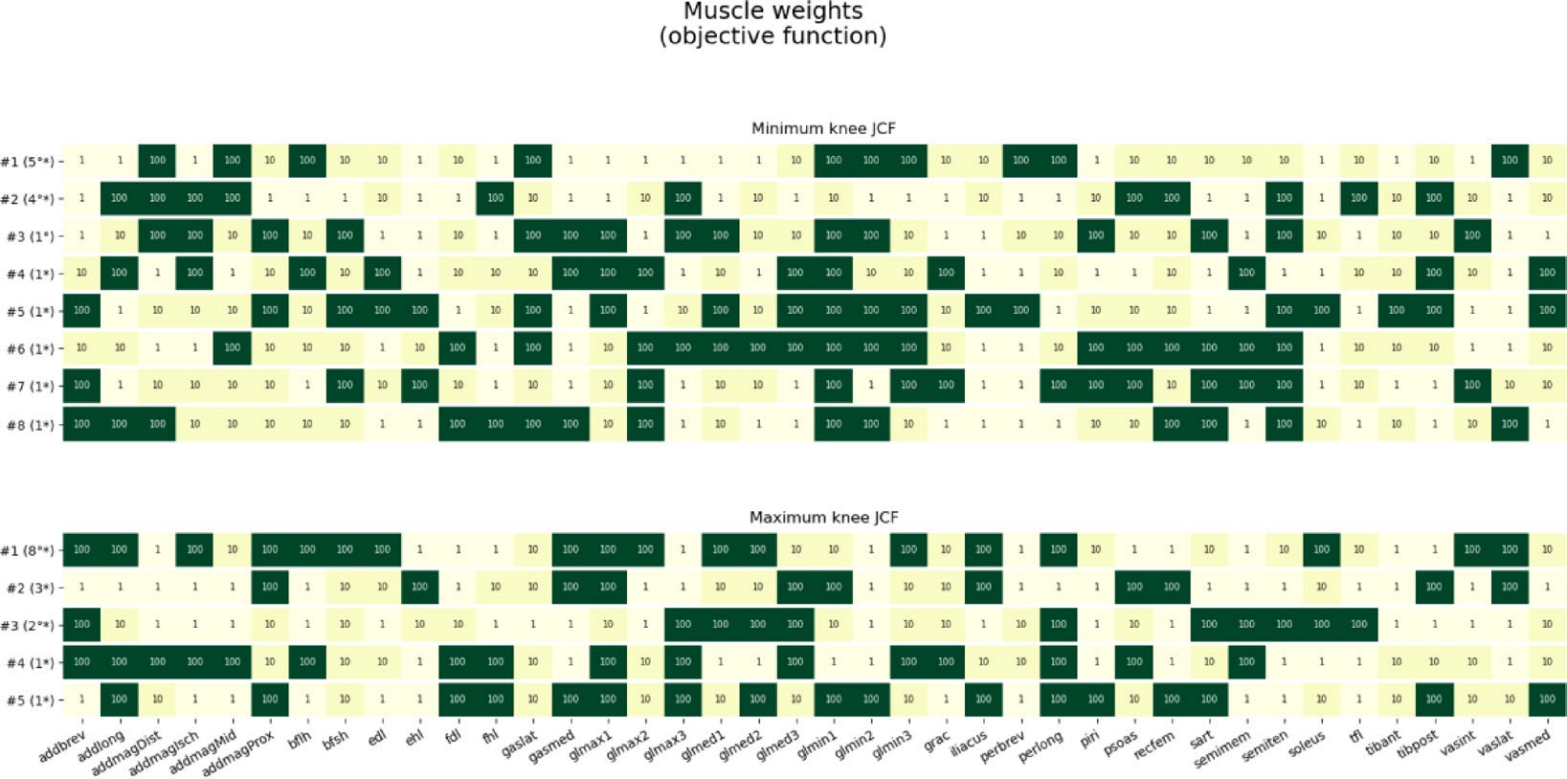
Combinations of weights associated with the minimal (top) and maximal (bottom) knee joint contact forces, across participants. The numbers in brackets indicate the number of subjects for whom the specific strategy was associated to minimal or maximal knee joint loading. * = healthy young adults, ° = elderly participants.

Looking at the strategies (set of weights) associated with the lowest and largest knee JCFs in the solution bands, a few clusters were identified (Fig. 9). To minimize the knee JCFs, two strategies were the most recurrent, across participants. Similarly, two combinations of weights (among the 10000 tested) emerged as those leading to the maximal knee JCFs. In this case, however, one combination of weights was associated with the largest knee JCF for most participants (8 out of 15, 4 FRE and 4 HYA).

## Discussion

The present study aimed (i) to explore how different neural strategies affect the knee joint contact forces during walking in healthy young and elder adults by means of computer modelling and simulation techniques, i.e., a static optimization approach and the MCMC method, and (ii) to study the effect of using experimental EMG data to inform the simulations. To this end, within OpenSim 4.3 and building on previous work^9,23^, we linearly scaled the Full Body Model^37^ to approximate the anthropometry of 15 individuals (10 young adults and 5 elders), which we employed to estimate a band of muscle forces and knee joint contact forces resulting from 10k different combinations of muscle activations obtained by changing the weight assigned to each individual muscle in the cost function. Additionally, for the cohort of young adults, a second set of simulations was performed, providing as input the available experimental EMG data^38^.

As expected, the use of EMG data to inform the simulations led to a marked reduction in the solution bandwidth (up to 69%), underscoring the benefits of informing MSK simulations with EMG data to achieve more physiologically plausible and interpretable estimates^17,39–43^. In addition, in most cases (Table 2), when the same sets of weights were applied, *EMG-constrained* simulations produced larger knee JCF predictions compared to *unconstrained* simulations. This is in line with previous work showing how the traditional static optimization approach, by discouraging muscle co-contraction, is likely to underestimate the predictions of an EMG-assisted approach, which more closely track experimental data^43^. What remains to be understood is whether the use of EMG data to simulate walking in populations presenting with abnormal gait patterns or less trivial tasks (possibly characterized by high variability) in healthy individuals would confirm these findings.

The comparison between young and elder adults (MCMC *unconstrained* simulations) revealed a difference in the solution bandwidth between populations, with the elderly showing a slightly larger range of variation across the entire stance phase at the knee (4.88 vs. 4.74 BW) and hip (3.28 vs. 3.37 BW) joints, but a narrower bandwidth at the ankle joint (1.43 vs. 1.65 BW). Similar conclusions could be drawn by looking at the solutions comprised between the 10th and the 90th percentiles, thus excluding the least recurrent (more unlikely) solutions. Due to the exploratory nature of the study, however, it remains unclear whether this slight increase in bandwidth is linked to an age-related deterioration in motor control, a mechanism that has been hypothesized and supported by several physiological and experimental studies^31,44–46^ and that may result in an altered joint kinematics. Of note, the cohort of elderly subjects in the present study consisted of highly active individuals^47^ with no history of musculoskeletal disorders or previous surgeries, who we can assume to have a good level of muscle control and whose kinematics did not significantly change from that of their younger counterparts^47^. To further explore the effect of ageing on muscle control, a longitudinal study on a larger cohort or a clinical ambulatory population would be needed. In addition, synergy-based methods could be exploited to further analyse the output solution band^48^ enabling an easier interpretation of the results, or even to reduce the complexity of the problem in the first place by reducing the number of possible combinations.

In line with previous work^8^, we observed that minimizing the knee JCF impulse (i.e., the area under the curve) led to an increase in the hip impulse (up to 31.7%) and to more modest variations at the ankle joint (on average, a 9.0 ± 6.0% increment compared to the minimum impulse identified among the 10000 simulations). Moreover, our results showed that the worst loading conditions at the knee (largest area under the JCF curve) are likely not associated to the most detrimental conditions at the hip and ankle joints. In fact, when considering the solution associated to the maximal knee JCF impulse, the hip and ankle impulses were markedly lower than their respective maximums within the MCMC band.

As expected, across both populations, the knee JCF solutions associated to the largest area under the curve reached higher peak values than the reference solution from static optimization (on average > 2.69 BW and > 4.71 BW, for the first and second peaks, respectively). On the other hand, the differences between the reference peak values and those from the solutions resulting in the minimal area under the knee JCF curve were slimmer (within ~ 2 BW), with the minimal solutions being generally lower than the reference solution. This finding underscores the fact that the solution identified via the classic implementation of the static optimization tends to sit in the lower end of the band of feasible solutions, but it is not necessarily associated to the minimum (peak) values.

Interestingly, although the most extreme loading conditions (lowest andlargest knee JCF in the solution band) resulted for many participants from the same strategy (i.e., set of weights in the cost function), our results showed how—even among young adults—different individuals may favour the activation of different muscle groups in the attempt to minimize or maximize the loads experienced at the knee joint. While this is not surprising among the elderly^46,49^, who may be influenced by an altered/reduced subjective perception of their strength or by the fear of falling^50^, it is counterintuitive in healthy young adults who perform a simple and repetitive motor task such as level walking. Certainly, the identification of potentially beneficial neural strategies can lead to the definition of more effective rehabilitation programs targeting—for example—the minimization of joint contact forces while preserving joint kinematics^51–53^.

This study has a few limitations that need to be considered. The small sample size, particularly for the elder cohort, does not allow for a generalization of the current results. This is due to the original design of the clinical study associated with this work and the narrow window for the data collection. Yet, we believe that the present findings represent a good starting point for any future (prospective) study aimed at assessing motor function in healthy adults and ageing populations. Second, the combinations of weights assigned to the muscles in the cost function to identify different neural strategies were inherited from Kainz et al.^23^, and not tailored to our participants. More specifically, each muscle was assigned a weight equal to 1, 10 or 100, as identified by the MCMC algorithm. As such, the assigned weights were not muscle-specific, possibly resulting in atypical or non-physiological muscle activations. Nonetheless, the presented approach (MCMC sampling method combined with weighted static optimization) allowed to identify a band of 10k solutions that minimized the overall sum of squared muscle activations while optimizing each individual muscle to a different extent (within three orders of magnitude), thus allowing to extensively explore the range of feasible solutions. Of note, all the identified solutions were mechanically feasible and the predicted muscle forces were within the physiological (tetanic) bounds. However, in the future, the use of EMG-informed or synergy-based weights should be explored to selectively look for combinations of weights reflecting synergistic muscle activations^54^ and to exclude non-physiological muscle behaviour. Third, in this study we used the solution bandwidth as a metric to quantify the variability of the solutions in response to different neural strategies (combinations of weights in the cost function) that produced the imposed kinematics. While computing the solution bandwidth may allow to better appreciate the effect of different control strategies on the joint loads, and possibly to shed lights on the effect of motor control variability^6,22^, this metric is not yet validated for the purpose. Therefore, we complemented our evaluation (1) by quantifying the difference between minimal/maximal solution and reference knee JCF solution in correspondence of the characteristic peaks, and the percent variation in muscle forces between the same cases (minimal and maximal knee JCF) and the reference solution, and (2) by qualitatively assessing the combination of weights associated to those extreme conditions. Last, we employed scaled generic models which may not accurately capture some subject-specific features of the human MSK system (e.g., muscle parameters such as tendon slack and optimal fiber length). However, the development of personalised MSK models, which could have partially tackled this issue (as some degrees of uncertainty on the definition of key parameters would have persisted) would have further increased the computational cost and overall time expenditure to complete the study (already quite substantial because of the 10k MCMC simulations per subject).

In conclusion, our findings preliminarily showed how ageing may be linked to an exacerbation of the consequences of suboptimal muscle control, as attested by the larger JCF solution bands (especially at the knee joint) observed among the elderly compared to the young adults when adopting different muscle activation strategies (i.e., combinations of weights in the cost function to be optimized). In addition, the present results underscore the importance of using EMG data to inform biomechanical computer simulations, to narrow down the solution space, towards more physiologically plausible estimates.

## Methods

### Experimental data and participants

The experimental data used in this study were previously collected as part of the Proto-Aging study^38,47^. The study was approved by the local Ethical Committee (Comitato Etico Area Vasta Emilia Centro: 152/2023/Sper/IOR), registered on the registry ClinicalTrials.gov (NCT05854316) and conducted in accordance with the Declaration of Helsinki. All participants signed a written informed consent prior to participating in the study.

The dataset hereby used is a subset of the Proto-Aging dataset, which included motion capture, ground reaction forces and surface EMG data from 20 healthy young adults and 5 elderly participants, collected while the participants performed a calibration trial (standing in T-pose) and a simple locomotor task, i.e. overground walking at self-selected walking speed^38^. A minimum of 10 walking trials, characterized by clean foot strike, were collected on all participants. The young volunteers were further asked to perform sit-to-stand, squat and stair ascend-descent tasks.

A total of 49 retroreflective markers (Figure S7, Supplementary Files) and 16 surface EMG sensors were respectively placed on pre-defined anatomical landmarks (as per lab procedures^55^ and the major lower limb muscles of both legs (9 on the dominant leg and 7 on the contralateral limb).

For this study, to reduce the overall computational cost, we selected a sub-set of the original dataset (15 subjects in total: 10 healthy young adults and 5 elderly participants. Table 4).

**Table 4:** Participants’ demographical data. The reported preferred walking speed (PWS) represents the mean walking speed recorded in the gait lab, for each participant.

Subject ID	Height (m)	Mass (kg)	Age (years)	Sex (F/M)	PWS (m/s)
HYA01	1.70	83	26.64	M	1.23
HYA02	1.60	54	27.41	F	1.16
HYA04	1.70	59	27.93	M	1.24
HYA05	1.77	68	27.12	M	1.22
HYA06	1.84	70	23.27	M	1.31
HYA10	1.77	77	33.57	M	1.27
HYA11	1.54	50	34.29	F	1.14
HYA12	1.63	50	23.07	F	1.38
HYA16	1.69	59	30.82	F	1.31
HYA17	1.92	82	22.21	M	1.45
FRE01	*1.73*	*80*	*70.13*	*M*	*1.22*
FRE02	1.80	78	64.96	M	1.36
FRE03	1.80	84	69.69	M	1.24
FRE04	1.70	62	66.40	F	1.35
FRE05	1.58	74	69.03	F	1.44
HYA (avg)	1.71 ± 0.11	65.2 ± 12.6	27.63 ± 4.22	4 F/6 M	1.27 ± 0.09
FRE (avg)	1.72 ± 0.09	75.6 ± 8.4	68.02 ± 2.25	2 F/3 M	1.32 ± 0.09

### Data processing

The experimental data were initially pre-processed in Vicon Nexus v2.12 (Vicon Motion Systems Ltd., Oxford, UK), to ensure that the marker trajectories were continuous and free from gaps, and that the marker labels had been correctly assigned. For each trial, the clean and synchronized data were then exported as a C3D file and further processed in MATLAB (v2020b) through MOtoNMS^56^.

More specifically, the EMG data were bandpass filtered (30–300 Hz, 2nd order Butterworth filter, double pass), rectified and low pass filtered (6 Hz, 2nd order Butterworth filter, double pass) to extract the linear envelopes, which were later normalized to the maximum value identified—for each muscle—among the available trials. Marker trajectories and force plates data were low-pass filtered at 8 Hz. Finally, the processed data were converted into a format compatible with OpenSim (i.e., marker trajectories as TRC, force plates and EMG data as MOT files).

For more details on the data processing steps, the reader is referred to^47^.

### MSK modelling and biomechanical simulations

Within OpenSim 4.3, the generic armless Full-Body model^9,37^ was linearly scaled to each participant’s size. The model comprised of 14 bodies, 20 degrees of freedom and 80 muscles (40 per leg)^1^. The maximal isometric forces of all muscles were scaled based on the ratio between the mass of the subject and that of the template generic model.

The scaled generic models were employed to perform biomechanical simulations of gait, exploiting the OpenSim API for MATLAB. Following an inverse approach, we first estimated the joint angles (inverse kinematics), then the external joint torques(inverse dynamics), musculoskeletal parameters (muscle analysis), muscle forces and activations (static optimization minimizing squared muscle activations), and joint contact forces (joint reaction analysis). Overall, 7 trials per participant were analysed, for a total of 105 (level walking) trials.

### Markov-chain Monte Carlo simulations

Following the steps outlined by Kainz et al.^23^ and Uhlrich et al.^9^, MCMC simulations were performed to assess muscle control variability during walking. This was achieved by assigning different weights to the muscles within the cost function to be minimized:

$${f_{cost}}=\mathop \sum \limits_{{i~=~1}}^{m} {w_i}{\left( {{a_i}} \right)^2}$$

where *m* is the number of muscles in the model, *a* is the muscle activation level and *w* the specific weight.

In total, 10,000 combinations of weights were tested, where muscles could be weighted 1, 10 or 100 (i.e., three different orders of magnitude). Of note, a simulation where all muscles are weighted 1 corresponds to a typical static optimization simulation which minimizes the overall sum of squared activations.

Due to the considerable computational cost associated with the simulations (on average 34 h, per subject), MCMC simulations were performed on one walking trial per participant. The selected trial was, for each participant, the trial for which the hip, knee and ankle joint kinematics more closely approximated the subject’s average values across the available trials.

A first batch of MCMC simulations where no experimental EMG data were provided as input, henceforth named *unconstrained*, were thus performed. At a later stage, a second set of MCMC simulations was performed, where experimental surface EMG data were used to inform the simulations. More specifically, constraints were added to the dynamic equilibrium problem to ensure EMG data tracking.

Due to the lack of EMG data from maximal effort tasks (e.g., squat, sit-to-stand) for the elderly population, which are crucial for proper normalization of the EMG linear envelopes, such *EMG-constrained* MCMC simulations were solely performed on data from the cohort of healthy young adults.

All simulations were run on a standard workstation (Intel Xeon E-2276G 3.80 GHz, 6 core, 64 GB RAM, on Windows 10 Pro, 64-bit).

### Data analyses

The predicted muscle forces and JCFs from OpenSim were interpolated to vectors of 101 points (to express the time-dependent variables as percentage of the stance phase, where the JCFs are non-negligible) and normalized to each subject’s bodyweight to enable comparisons among individuals.

The knee JCF profiles were initially visually inspected to identify any abnormal behaviour (e.g., excessive values or discontinuities). A quantitative assessment followed, (1) to determine the bandwidth of the solution space when the MCMC approach was implemented and (2) to detect and quantify any differences between the *unconstrained* and *EMG-constrained* MCMC simulations.

The solution bandwidth was calculated both in correspondence of the two characteristic peaks (of the vertical component of the ground reaction forces) and across the entire analysed windows, as the difference in magnitude between the highest and lowest values. In addition, the predicted JCFs were sorted and organized in quantiles, to identify the most recurrent values across the 10k solutions (i.e., 10th to 90th percentile). For completeness, the latter analysis was also conducted on the JCFs predicted at the hip and ankle joints. Furthermore, to assess the effect of maximising/minimising the knee JCF on the load experienced by the other joints, for each participant, we computed the percent difference between the area under the hip/ankle JCF solution associated to the maximum and minimum knee JCFs and the corresponding maximal/minimal area under the hip or ankle JCFs.

To evaluate the effect of the EMG constraint on the knee JCF solutions, for each HYA subject, we first computed the ratio between the solution bandwidth resulting from the *EMG-constrained* and *unconstrained* simulations, using the *unconstrained* values as reference:

$$bandwidth~ratio=bw/b{w_{unconstrained}}$$

where *bw* is the average bandwidth across the stance phase of the gait cycle for either the *EMG-constrained* or *unconstrained* simulations.

Then, we calculated the relative area difference (RAD) between each *unconstrained* and corresponding (i.e., with the same set of weights) *EMG-constrained* simulation. The latter analysis, which was solely performed on the data from the healthy young cohort (see *Markov-chain Monte Carlo simulations* section), was repeated for all 10,000 combinations of weights, and the mean value per participant was then extracted. A positive RAD value means a larger area under the knee JCF curve when EMG data are provided as input; vice versa for negative RAD values.

In addition, for the MCMC *unconstrained* simulations, we evaluated the estimated forces generated by the ten primary muscles crossing the knee joint (i.e. Vastus lateralis—VL, vastus medialis—VM, Rectus femoris—RF, Bices Femoris—BFL, Sartorius—SART, Tensor Fascia Lata—TFL, Semimebranosus—SM, Semitendinosus—ST, Lateral Gastrocnemius—LG, Medial Gastrocnemius—MG). More specifically, we computed the cumulative area under the curve (i.e., the impulse) resulting (1) from the static optimization solution (with all muscle weights set to 1, hereby used as reference), and (2) from the best and worst MCMC simulations (corresponding to the cases where the knee JCFs were respectively the smallest and the largest in the solution band, among the 10000 simulations). The percent variation with respect to the static optimization solution was thus extracted for all muscles and subjects, and reported in boxplots for a qualitative assessment. For the same conditions (minimal and maximal area under the knee JCF profile) we further computed, in correspondence of the two characteristic peaks, the distance from the reference solution.

Last, for all subjects, we extracted the weight assigned to each muscle for the solutions associated with the largest and smallest knee JCF, and generated a heatmap to appreciate the various strategies adopted by the participants to maximise or minimize the knee joint loads.

Of note, as the use of EMG data was associated with some noise and artifacts at the beginning of the stance phase, all the analyses performed to compare *EMG-constrained* and *unconstrained* simulations were conducted on a narrower window (i.e., between the two characteristic peaks of the vertical component of the ground reaction forces measured by the force plates, not on the entire stance phase).

### Statistical analysis

A Wilcoxon signed rank test was performed to determine whether the differences in knee JCFs bandwidth observed between *unconstrained* and *EMG-constrained* simulations in the HYA cohort were statistically significant. The same test was also performed to compare the RAD values, and to identify any significant differences in the mean solution bandwidths across the stance phase of the gait cycle between populations. In both cases, the significance level (p-value) was set to 0.05. For the remaining sets of results and analyses, only descriptive statistics was used, due to the limited size of the elderly cohort.

## Supplementary Information

Below is the link to the electronic supplementary material.

Supplementary Material 1

## Data Availability

The input data and models used to perform the Monte Carlo simulations are available at: (https://github.com/GiorgioD89/ProtoAgingMCMC). The experimental data associated to this work, and collected as part of the ProtoAging study^48^, are stored on Zenodo and may be made available upon reasonable requests to the author: (https://doi.org/10.5281/zenodo.15100077) (Proto-Aging data collection).

## Footnotes

1 adductor brevis, adductor longus, adductor magnus (distal, ischial, middle, proximal), biceps femoris long head, biceps femoris short head, extensor digitorum longus, extensor hallucis longus, flexor digitorum longus, flexor hallucis longus, gastrocnemius lateralis, gastrocnemius medialis, gluteus maximus (superior, middle, inferior), gluteus medius (anterior, middle, posterior), gluteus minimus (anterior, middle, posterior), gracilis, iliacus, peroneus brevis, peroneus longus, piriformis, psoas, rectus femoris, sartorius, semimembranosus, semitendinosus, soleus, tensor fascia lata, tibialis anterior, tibialis posterior, vastus intermedius, vastus medialis, vastus lateralis.

## Code availability

The Monte Carlo simulations implementing the unconstrained and EMG-constrained Static Optimization are based on the codes by Uhlrich et al.^9^ and Kainz et al.^23^, which can be found at the following links: https://github.com/stanfordnmbl/MatlabStaticOptimization and https://github.com/HansUniVie/MuscleCoordinationRetraining. Additional scripts hereby employed to analyse the results are available at: https://github.com/GiorgioD89/ProtoAgingMCMC.
