# Supplementary material for: Exploring the effect of different neural strategies on the knee joint contact forces during walking in adults
Source: Sci Rep. 2026 Apr 9;16:16676. doi: 10.1038/s41598-026-46419-8 (PMC13219765; doi:10.1038/s41598-026-46419-8)
Supplement: Supplementary file 1 — Supplementary Material 1 [file 41598_2026_46419_MOESM1_ESM.docx]

# SUPPLEMENTARY FILES

# EXPLORING THE EFFECT OF DIFFERENT NEURAL STRATEGIES ON THE KNEE JOINT CONTACT FORCES DURING WALKING IN ADULTS

Giorgio Davico^1^, Enrico Toccaceli^1^, Luciana Labanca^2^, Maria Grazia Benedetti^†,2,3^, Marco Viceconti^1,4^

^1^ Department of Industrial Engineering, Alma Mater Studiorum - University of Bologna (IT)

^2^Physical Medicine and Rehabilitation Unit, IRCCS Istituto Ortopedico Rizzoli, Bologna (IT)

^3^Department of Biomedical and Neuromotor Sciences, Alma Mater Studiorum - University of Bologna (IT)

^4^Medical Technology Lab, IRCCS Istituto Ortopedico Rizzoli, Bologna (IT)

CORRESPONDING AUTHOR:

Dr. Giorgio Davico

Department of Industrial Engineering

Alma Mater Studiorum - University of Bologna

Viale del Risorgimento 2, 40136 Bologna (IT)

Email: [giorgio.davico@unibo.it](mailto:marco.viceconti@unibo.it)

*
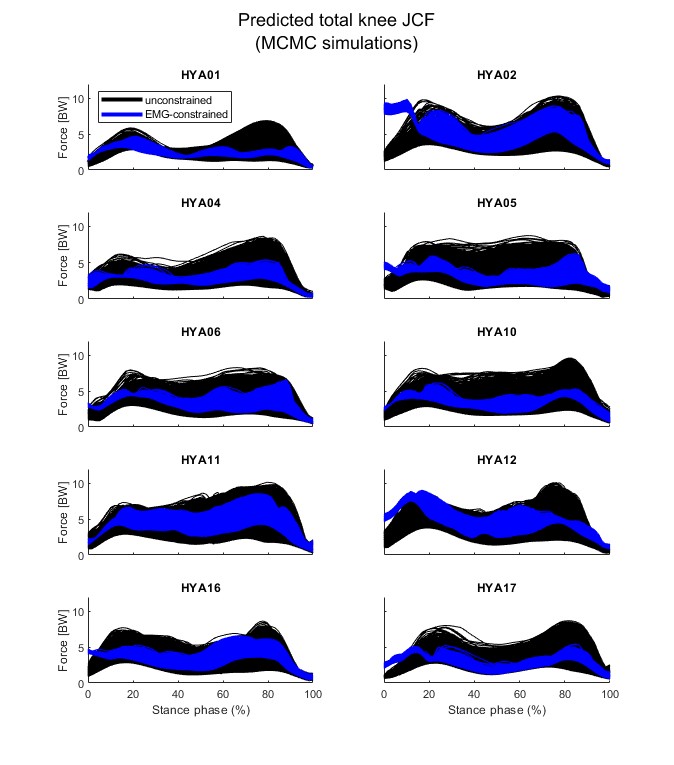
Figure S1 Knee JCFs predicted by the unconstrained (black) and EMG-constrained (blue) simulations, for the 10 young adults, normalized to each participants’ body weight.*

*
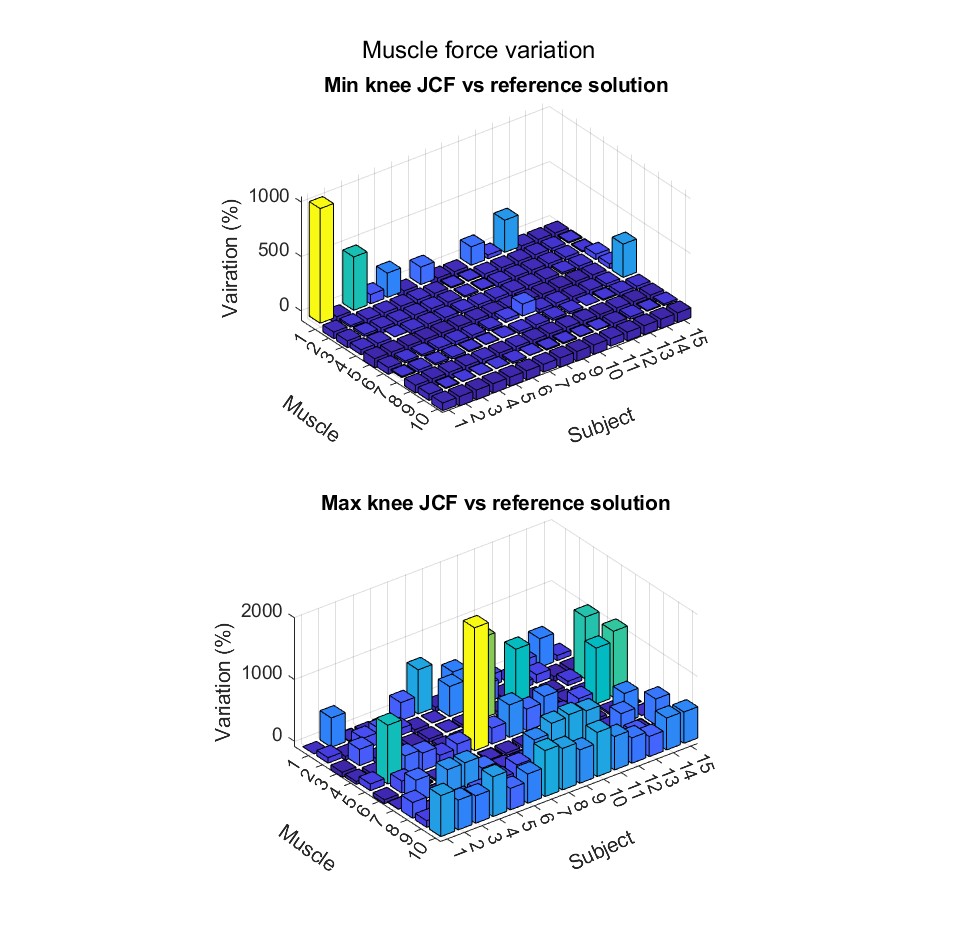
Figure S2 Bar plot showing the percent variation in muscle force for the ten primary muscles spanning the knee joint resulting from the comparison between the solution corresponding to the minimal (top) or maximal (bottom) knee joint contact force and the reference solution, for all subjects. Muscle numbers: 1 = RF, 2 = VL, 3 = VM, 4 = BFL, 5 = SM, 6 = ST, 7 = LG, 8 = MG, 9 = SART, 10 = TFL. The first five subjects correspond to the elderly subjects.*

*
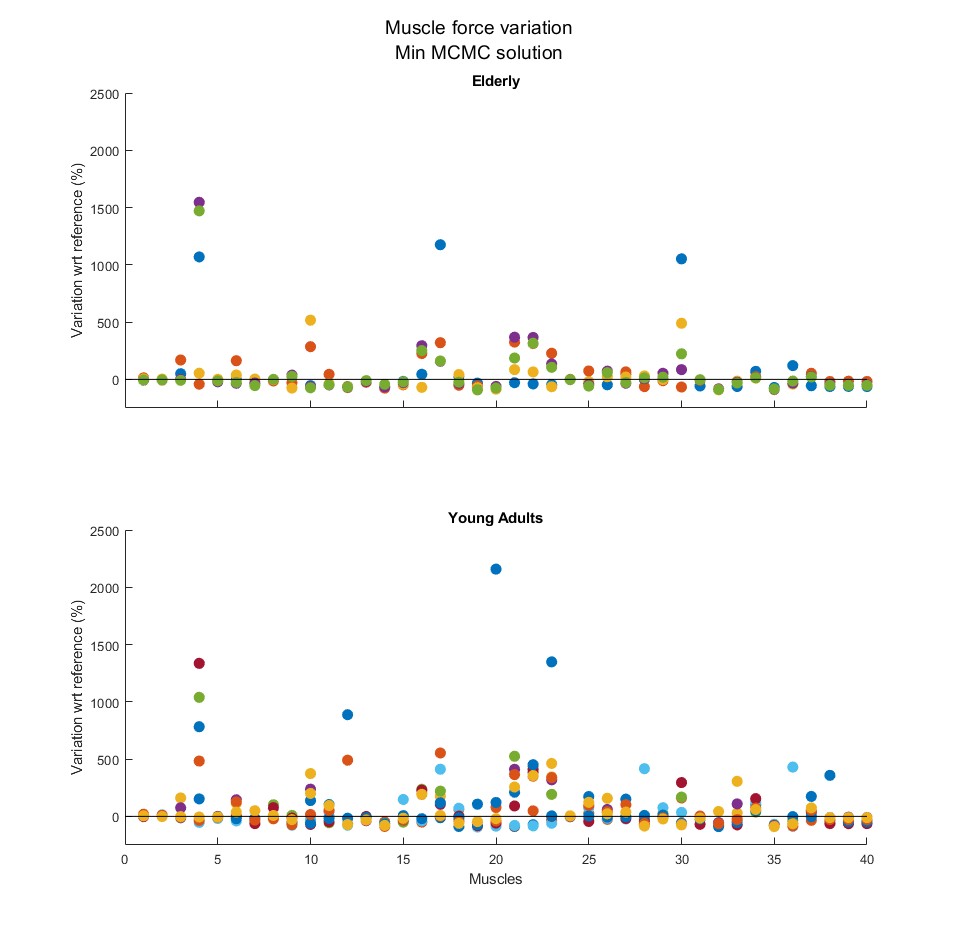
Figure S3 Scatter plot showing the variation in force for the muscles of the dominant leg between the reference static optimization solution and the solution associated to the minimal knee JCF, for the elderly (top) and young participants (bottom). Different colours refer to different participants. Muscles, on the x axis, are displayed in alphabetical order according to OpenSim naming convention: 1-6 (hip ab/adductors), 7-8 (biceps femoris), 9-12 (extensor/flexor toes), 13-14 (gastrocs), 15-23 (glutei), 24 (gracilis), 25 (iliacus), 26-27 (peronei), 28 (piriformis), 29 (psoas), 30 (rectus femoris), 31-33 (hamstrings), 34 (soleus), 35 (tfl), 36-37 (tibialis), 38-40 (vasti).*

*
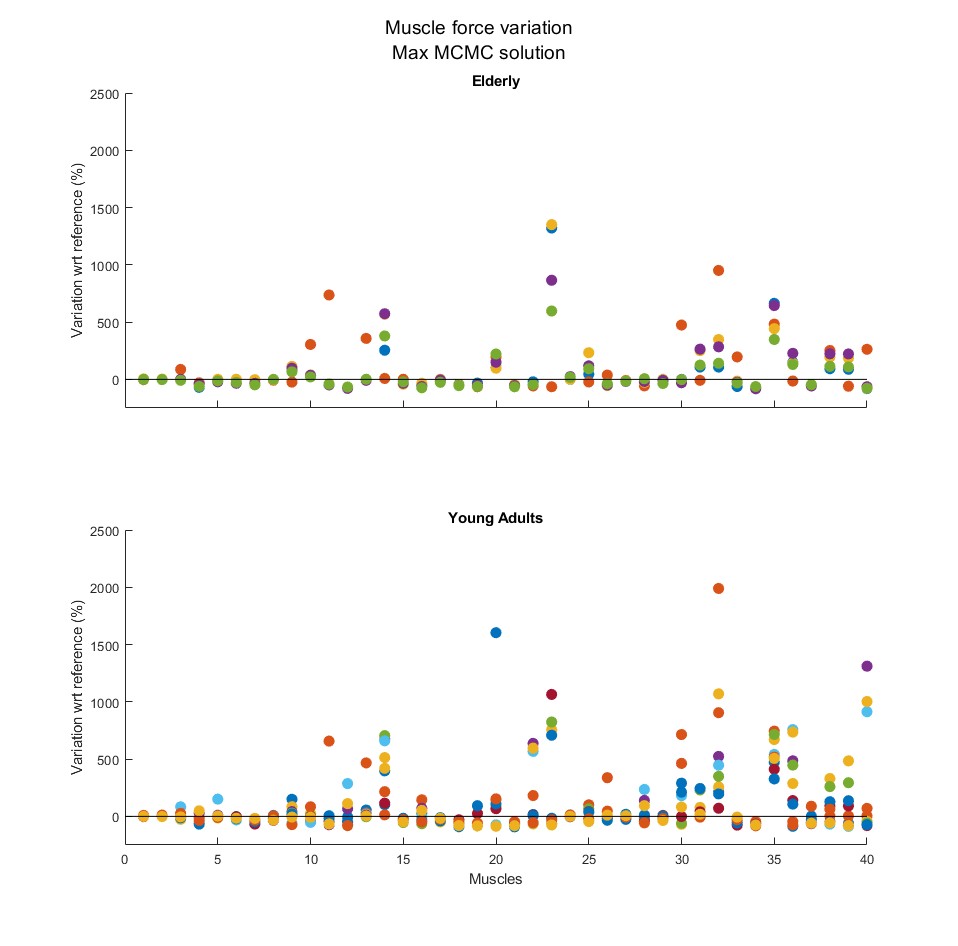
Figure S4 Scatter plot showing the variation in force for the muscles of the dominant leg between the reference static optimization solution and the solution associated to the maximal knee JCF, for the elderly (top) and young participants (bottom). Different colours refer to different participants. Muscles, on the x axis, are displayed in alphabetical order according to OpenSim naming convention: 1-6 (hip ab/adductors), 7-8 (biceps femoris), 9-12 (extensor/flexor toes), 13-14 (gastrocs), 15-23 (glutei), 24 (gracilis), 25 (iliacus), 26-27 (peronei), 28 (piriformis), 29 (psoas), 30 (rectus femoris), 31-33 (hamstrings), 34 (soleus), 35 (tfl), 36-37 (tibialis), 38-40 (vasti).*

*
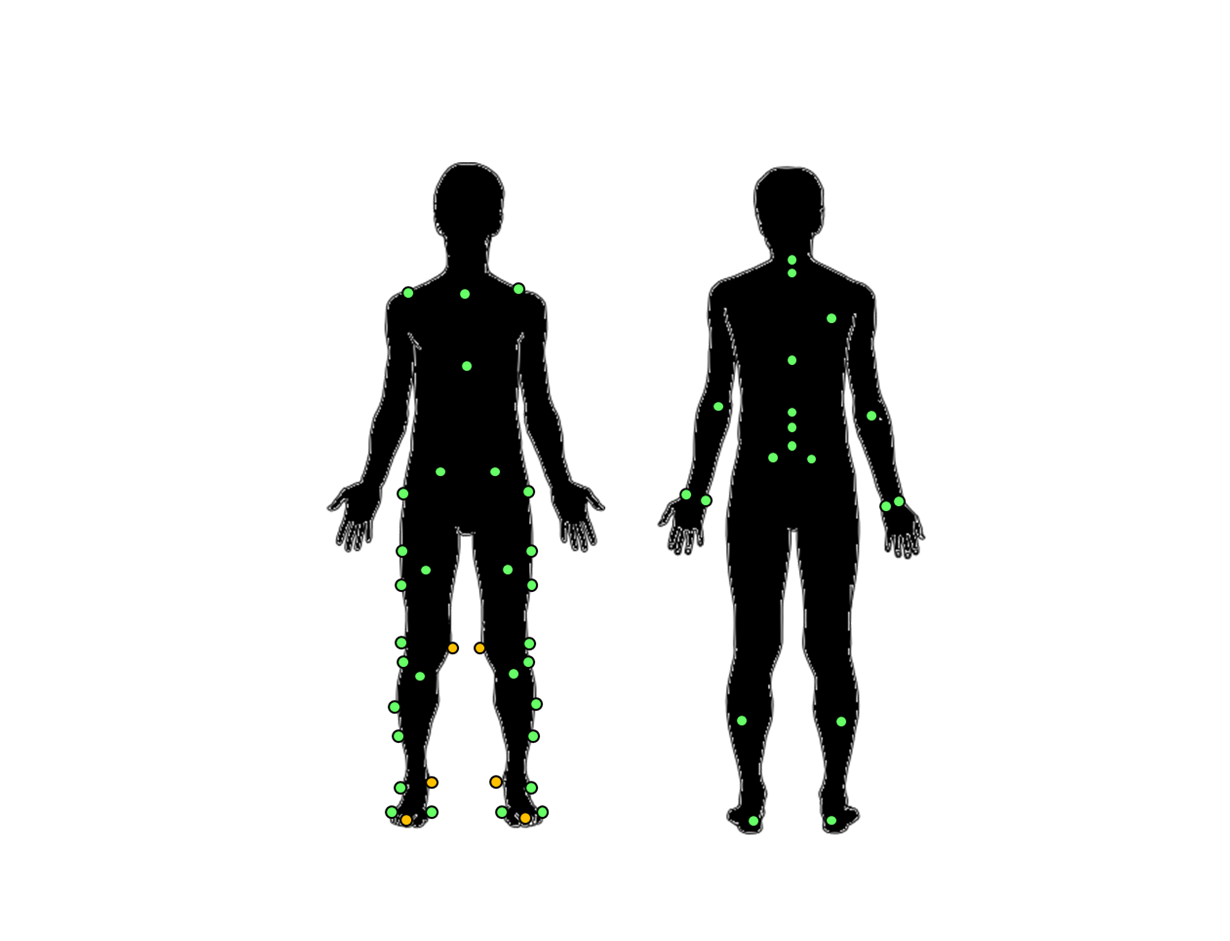
Figure S5 Markerset employed for the study. Overall, 55 reflective markers were placed on anatomical landmarks on both upper and lower limbs. The six orange markers on the left were remove after calibration.*
